# An interactive retrieval system for clinical trial studies with context-dependent protocol elements

**DOI:** 10.1371/journal.pone.0238290

**Published:** 2020-09-18

**Authors:** Junseok Park, Seongkuk Park, Kwangmin Kim, Woochang Hwang, Sunyong Yoo, Gwan-su Yi, Doheon Lee

**Affiliations:** 1 Department of Bio and Brain Engineering, Korea Advanced Institute of Science and Technology (KAIST), Daejeon, Republic of Korea; 2 Bio-Synergy Research Center, KAIST, Daejeon, Republic of Korea; 3 Information & Electronics Research Institute, Daejeon, Republic of Korea; 4 The Milner Institute, University of Cambridge, Cambridge, United Kingdom; 5 School of Electronics and Computer Engineering, Chonnam National University, Gwangju, Republic of Korea; Fayoum University Faculty of Computers and Information, EGYPT

## Abstract

A well-defined protocol for a clinical trial guarantees a successful outcome report. When designing the protocol, most researchers refer to electronic databases and extract protocol elements using a keyword search. However, state-of-the-art database systems only offer text-based searches for user-entered keywords. In this study, we present a database system with a context-dependent and protocol-element-selection function for successfully designing a clinical trial protocol. To do this, we first introduce a database for a protocol retrieval system constructed from individual protocol data extracted from 184,634 clinical trials and 13,210 frame structures of clinical trial protocols. The database contains a variety of semantic information that allows the filtering of protocols during the search operation. Based on the database, we developed a web application called the clinical trial protocol database system (CLIPS; available at https://corus.kaist.edu/clips). This system enables an interactive search by utilizing protocol elements. To enable an interactive search for combinations of protocol elements, CLIPS provides optional next element selection according to the previous element in the form of a connected tree. The validation results show that our method achieves better performance than that of existing databases in predicting phenotypic features.

## Introduction

According to a recent report, the clinical cost per approved new drug has increased to $2.9 billion [1]. Another report also indicated that the global clinical trial market is estimated to be worth $68.9 billion and grow at a compound annual growth rate of 5.7% by 2027, owing to high demand for treatment of chronic diseases [2]. Clinical trial protocols are essential for the ultimate success of trials [3]. Well-established and optimized protocols are warranted to improve adherence to clinical procedures, help avoid unnecessary protocol amendments, facilitate preliminary assessments of latent issues, and reduce the risk of trial failure, thereby contributing to the success of clinical trials [4, 5]. Poor study design may lead to constraints on subject participation, dropout, and early termination, resulting in increased cost burdens and loss of efficiency [6, 7]. Moreover, it results in protocol amendments, some of which may be avoidable [8]. This has brought about an unprecedented demand for computerized systems designed to assist many stakeholders (i.e., pharmaceutical companies, clinical research organizations, health authorities, ethical committees or institutional review boards, courier vendors, and academic medical centers), aimed at making specific and deliberated choices and developing standardized protocols [9, 10]. Although, it is well known that clinical trials should be supported by rigorously developed protocols, there remains limited published evidence or guidance on the development of clinical trial protocols [11]. The majority of protocols still adhere to expert guidelines combined with scientific evidence and expert clinicians' opinions; thus, limiting the amount of knowledge available that can be used to underpin the development of cost-effective and efficient protocols.

The advantage of using computerized systems is that they are quick and allow iterated searches for previous protocols related to the study of interest. In this regard, computerized systems used to retrieve reliable protocols should have a database system as the baseline feature set and an advanced information retrieval method. The database system is a set of systematically managed data repositories [12]. It could include information analysis to find features for accurate search. The information retrieval method consists of query analysis, information analysis, and relevance calculation between query and information [13]. The method we propose in this study addresses only the database system; the information retrieval method is the next step of our study.

From the database system perspective, the previous approaches can be grouped into two categories: expert guidelines and computerized systems. Computerized systems can be subdivided further into database and automated systems.

First, existing expert guidelines help researchers design their own trial protocols [14]. Although referring to expert opinions guarantees the credibility of the protocol design, there are two obvious limitations: the protocol can only be applied if credible guidelines exist in that particular clinical field and not all guidelines offer specific values for all elements of trial design. Consequently, the determination of these specific elements relies on the subjective intuition of individual researchers.

Second, computerized system approaches offer an automated method for designing a protocol. For example, a context-aware architecture has been developed for clinical trial protocol design composed of a decision support module and semantic search engine [15]. Although the idea of constructing an automated system was an innovative approach when it was proposed, this system offers only limited performance. For instance, the idea focuses on creating scientific queries for finding information about a clinical trial protocol and retrieving only related papers through queries. Furthermore, the web-based service is no longer available.

Current state-of-the-art systems are computerized and use a database of clinical trial protocols. These databases contain extensive information on previous clinical trials and clinical experiences covering a wide range of clinical fields [16–18]. Researchers can retrieve information from specific clinical trials according to their purpose. However, current clinical trial databases offer only limited support in searching for clinical trial protocols. The clinical trial database system uses medical subject heading (MeSH) terms but does not cover all of the text in the protocols, preventing a truly semantic search [16]. Moreover, the current databases do not allow structural protocol searches to retrieve context-dependent protocol elements. A biomedical literature database system (PubMed) could be used to search for clinical trial protocols [19]. However, this would not be efficient because additional work would be required to extract the necessary information from the retrieved literature.

**Fig 1:**
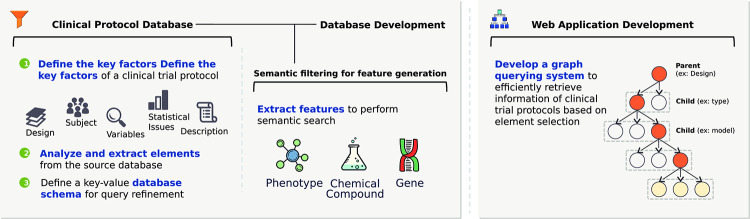
Overview of CLIPS development process.

To overcome the limitations of the current database systems, we present a clinical trial protocol database system (CLIPS) that enables a semantic search for the core content of clinical trial protocols, along with filterable semantic features and frame structures for the protocols. In detail, we collected essential data and defined the structure of protocols from a public database of clinical trials [20]. We used a text mining pipeline based on Metamap, Moara, and Chemspot to understand the contextual meaning of texts in clinical trial protocols and to increase search accuracy [21–24]. To resolve the difficulty of retrieving specific protocols from a database of complex structures, we developed a graph-based querying system (Fig 1).

## Definitions

Clinical trial protocols consist of several elements that can be grouped into factors according to their characteristics [25]. We define the terms “element” and “factor” as follows.

- Element: individual items constituting the clinical trial protocol. An element has a value that defines the protocol. For example, “model” is an element, and the value of this element can be “crossover.”
- Factor: a common characteristic of grouped elements. A factor can have multiple elements. For instance, “model” and “allocation” elements are used to design a protocol. Thus, they belong to the “design” factor. Another example is the “enrollment type” and “gender” elements which determine the subject of a protocol and are part of the “subject” factor.

For instance, the “design” factor of a protocol includes 14 elements, and among the elements, the “model” contains 12 values. The values consist of “crossover assignment” to “case-only”, and the “model” element of the “design” factor has one of the values. S1 Fig shows the above example.

## Related work

### Guidelines

The retrieval of documents containing the contents of a protocol design is one method of determining a clinical research protocol. The document containing the guidelines covers the overall information on clinical research protocols. This is the most basic approach for gathering information to develop a protocol. Chan et al. [3] proposed SPIRIT, which is a high-quality guideline containing 33 checklist items for the development of a clinical trial protocol. Meeker-O’Connell et al. [26] developed a principle document that defines the factors needed to assure patient safety and reliability in a trial. Moreover, some guidelines specify protocols for examining the efficacy of food or food components for specific diseases [27]. For instance, documents describing gut health and immunity, diet-related cancer, and atherosclerosis are included [28–30]. However, guideline-based approaches use subjective judgment in determining the information to be included [31]. This limitation can result in different outcomes depending on the user.

### Database systems

Database-based information retrieval systems can be utilized to retrieve clinical protocols. Zarin et al. developed clinicaltrials.gov, the largest database retrieval system, by collecting all published clinical trial documents, including regulatory mandates and a broad group of trial sponsors [18]. Tasneem et al. established and operated a relational database containing all clinical trials registered with clinicaltrials.gov [16]. Furthermore, systems for protocol retrieval use general document retrieval technologies, e.g., PubMed, Scopus, Web of Science, and Google Scholar [32–35]. However, current database-based retrieval systems have limited ability for protocol-specific search objectives, such as retrieving the protocol structure or sequentially selecting context-dependent protocol elements.

### Intelligent systems

Intelligent systems are an effective approach for retrieving clinical trial protocols. Tsatsaronis et al. developed an intelligent system based on a context-aware approach for automated protocol design [15]. Their system supports study- and domain-driven searches. Study-driven searches use the parameters (i.e., condition, intervention) of a particular trial as provided by a researcher. In domain-driven searches, a researcher selects options according to the study domain; the system then automatically searches and categorizes the retrieved information. However, a system such as this is currently not accessible. We assume that the author terminated the system.

## Methods

We developed a clinical trial protocol database system. To accomplish the objective, we proposed step-by-step methods including database development, semantic feature generation, and a web-based retrieval system. We constructed the database from a public database of clinical trials and organized essential data to reflect the structure of protocols. Semantic feature generation is a core part of the clinical trial protocol retrieval system. We generated filterable semantic features to offer context-specific searches for the protocols from the original text based on named entity recognition tools. The semantic features consist of phenotypes, genes, and chemical compounds. Finally, we made a web-based protocol retrieval application. Text-based search cannot scrutinize the complex structure of the protocols. Therefore, we devised a graph-based search interface as a query refinement method.

### Database development for a protocol retrieval system

A clinical trial protocol presents the structure of a clinical trial and is composed of various elements that can be clustered into key factors. In this study, we defined five key factors based on a previous baseline research project [25]: design, subject, variables, statistical issues, and descriptions. The design factor determines how the trial is structured and modeled to measure data generated during the trial. The subject factor determines who is eligible to participate in the trial and how they are treated to ensure the generalizability of the target population. The variables are the parameters to be measured to evaluate the efficacy or safety of a drug or treatment. The statistical issues describe how the clinical trial will be analyzed, specifying sampling procedures or statistical significance. Finally, the description factor covers additional information such as the organization, different phases, and additional explanations of the protocol or trial itself.

We selected and clustered elements from Aggregate Analysis of ClinicalTrials.gov (AACT), which was released on March 27, 2015 [16]. To do this, we first downloaded a dump file of the AACT database and completely overhauled the loaded database to obtain 42 tables of 270 columns. Then, we classified the data types into four elements: categorical, value, description, and not union (N/U). Categorical-type elements contain categorical variables; the sequential selection of these elements can determine the protocol structure (S1 File). Value-type elements include interval and ratio data that are important values in key factors. Description-type elements contain additional explanatory text, numeric values, and abbreviated words or dates for the description factor. The N/U-type element consists of primary keys, foreign keys, and database management values.

We clustered the categorical and value types into the pre-defined five key factors according to the above-mentioned criteria, including design, subject, variable, and statistical issue factors (Table 1).

**Table 1:** Key factors and their elements in CLIPS.

Key factors	Categorical Type Elements	Value Type Elements
**Design**	type, model, allocation, time perspective, masking, masked role, primary purpose, endpoint classification, group type, intervention type	number of groups, design group, group label, description, intervention name, intervention other name, intervention desc
**Subject**	enrollment type, gender, is health volunteers, minimum age unit, maximum age unit	enrollment, minimum age, maximum age, study population, target population, criteria
**Variable**	variable group, safety issue, measure type, dispersion, measure type	biospec descry, biospec retention, measure, time frame, description, unit of measure, category title
**Statistical Issue**	sampling method, variable group, dispersion type	population, measure, param type, dispersion type, dispersion value, statistical method, ci percent, ci lower limit, ci upper limit, ci n slides, categorical title, baseline value, spread, lower limit, upper limit

We designed a table schema and amassed the data compilation progress. The N/U elements were eliminated because we constructed a relational table with key-value attributes, discarding unnecessary keys and values for database management. The use of key-value attributes makes it possible to search the skeleton structure of a clinical trial protocol efficiently and effectively manage the data storage for deploying inconsistent data [36]. We designed a table schema accounting for these aspects (Table 2). The next step was data compilation. We organized the element values by resolving typographical errors and reflecting dependency structures. Then, we removed some control characters and type conversions. Furthermore, we removed ambiguous design types, which are null, and expanded the access and observations (patient registry) to search for specific clinical trial protocols. As a result, we collected 184,634 clinical trial protocols and their detailed information. The resulting database can be used to optimize query refinement for retrieving protocol information.

**Table 2:** Table schema for clinical trial protocol.

Column Name	Column Type
'ClinicalTrialID	Char(10)
Design	JSON
Subject	JSON
Variable	JSON
Statistical Issue	JSON
Description	JSON

### Semantic filtering feature generation

Although we developed a clinical trial protocol database by using a frame structure for all protocols to have a similar structure, frame structure similarity does not guarantee similarity of the detailed protocol content. MeSH offers a potential solution and is used for indexing and cataloging clinical trials in ClinicalTrials.gov and AACT [16, 27]. However, MeSH has limited coverage that does not extend across the spectrum of various biomedical terminologies [37]. To solve this limitation, various biomedical semantic features were extracted to find or filter similar clinical trials in the structure being searched.

**Fig 2:**
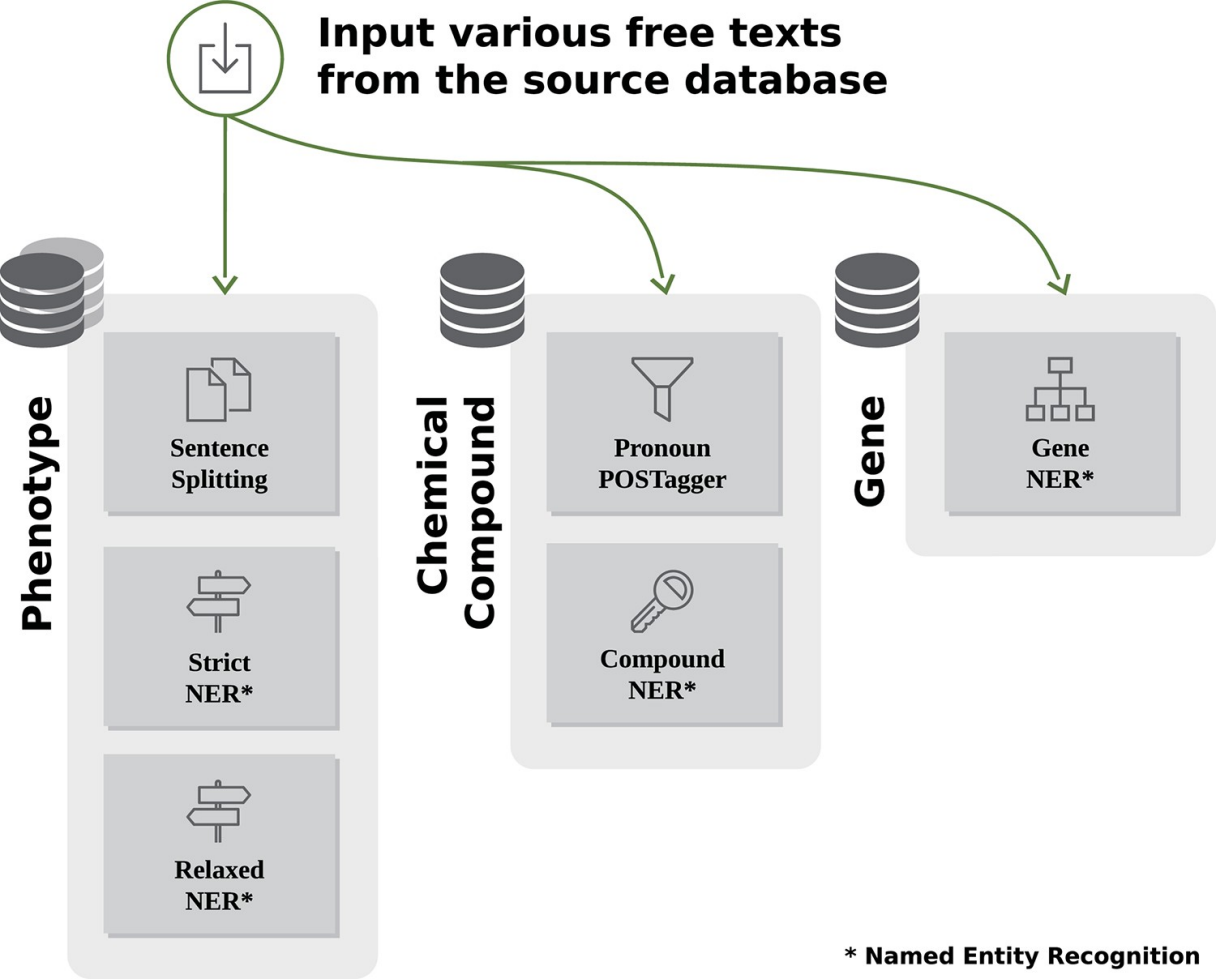
Generation of data for semantic search.

We generated filterable semantic features related to the conditions and interventions that are considered significant in clinical trials, resulting in a subdivided semantic similarity search. The condition was a phenotype, including any diseases and disorders, observed during clinical trials as well as reported symptoms. The disease-specific phenotype is a set of observable characteristics. Drugs commonly refer to interventions that are the focus of clinical trials; they can involve chemical compounds [38]. Similar clinical trials can be searched for or filtered using each of the corresponding elements. In addition, the identification of similar target genes or proteins is a potential method of searching for similarities among chemical compounds and phenotypes as they are a molecular proxy that links them [39, 40]. Thus, we applied named entity recognition (NER) to the phenotypes, chemical compounds, and genes to enable a semantic search, for which semantic filters were employed for the following description elements: brief title, official title, brief summary, detailed description, keywords, and conditions (Fig 2).

#### Phenotype

We extracted semantic features to represent disease-specific phenotype terminology. The unified medical language system (UMLS) is a repository of integrated biomedical terminologies, and thus, we used UMLS2015AB to process phenotype words [37]. To employ NER on descriptive values, we applied Metamap 2016 and cTakes 3.2.2 [41, 42]. We combined each result and removed duplicates using the above-mentioned tools to synthesize the advantages [43]. Next, we selected 15 semantic types, which are considered disease phenotypic types, and removed the other types from the results (Table 3). As a result, disease phenotypic features with unique concept IDs were generated for each clinical trial.

**Table 3:** Selected phenotypic types from UMLS.

Entity Type ID (TUI)	Entity Type Name
T038	Biologic Function
T039	Physiologic Function
T041	Mental Process
T019	Congenital Abnormality
T020	Acquired Abnormality
T033	Finding
T034	Laboratory or Test Result
T046	Pathologic Function
T047	Disease or Syndrome
T048	Mental or Behavioral Dysfunction
T049	Cell or Molecular Dysfunction
T184	Sign or Symptom
T190	Anatomical Abnormality
T191	Neoplastic Process
T037	Injury or Poisoning

#### Chemical compound

We applied NER to chemical compound entities from the descriptions given by ChemSpot [44]. ChemSpot provides Chemical Abstract Service (CAS) IDs and International Chemical Identifiers (InChI) but does not provide standard InChIKeys. The InChIKey is the compacted version of InChI, and the standard InChIKey is a stable identifier for reflecting the identifier version designation [45]. Moreover, standard InChIKeys are considered to provide equivalent descriptions between compounds in drug discovery [46]. To take advantage of the standard InChIKeys for chemical compound entities, we examined the original words of the NER-processed entities by using ChemSpider [23]. ChemSpider’s simple application programming interface (API) was an advantage that allowed us to generate chemical compound entities with standard InChIKeys, InChIs, and the simplified molecular-input line-entry system notation.

### Gene

We appended gene entities in the elements of semantic filters. The gene annotation tool, Moara, was used for gene NER, considering that Moara can perform both recognition and normalization of gene entities, recognizing entities and their positions in the input text, and linking the entities to gene IDs in a known gene database [47]. Moara provides various preconstructed machine learning models for certain organism species. For our task, we adopted the human-oriented model. For gene normalization, we obtained lists of gene IDs corresponding to each gene entity. The gene ID with the highest score was selected and mapped to the recognized gene term.

### Web application development for query refinement

The structures of clinical trial protocols have become increasingly complex [48]. The complexity of the protocol level is inversely related to clinical trial performance, as complex protocols negatively impact factors such as protocol amendment rates, patient recruitment, and retention rates [49]. In addition, the increasing complexity of the protocols hinders the design of new protocols because clinicians referring to previous clinical trials to design a protocol inevitably face difficulties in searching for suitable examples. Thus, from a query refinement standpoint, we developed the CLIPS web application to provide a graph querying interface for retrieving information about reliable clinical trial protocols, rather than a text querying interface that cannot visualize the dependency among prior elements affecting protocol structure [50, 51].

**Fig 3:**
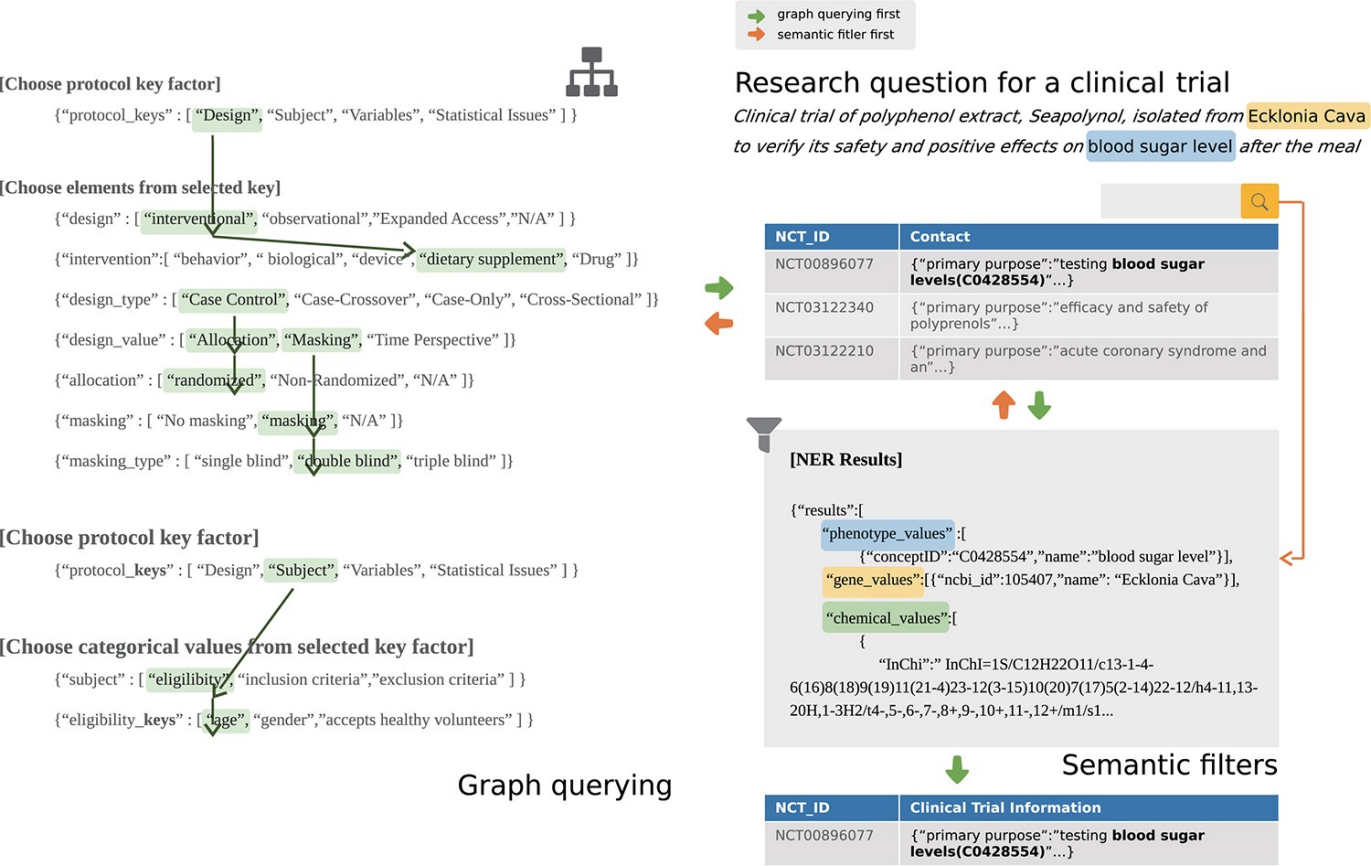
Example of background data-flow on CLIPS from a research question in a clinical trial.

We defined categorical-type elements as the frame structures of protocols. Although we have provided default orders of the elements, the user is free to choose the order. Once a decision about the order has been made, the user can find varying combinations of protocol elements via the graph-based search interface. Dependent elements are retrieved from the database in real-time, and the user can confirm the number of existing protocols corresponding to selected elements. The user also can search for other protocol frame structures after clipping the selected structure to the user clip pane. To search for complete information about the selected protocol structures, the user must click on a clipped protocol to examine the details of the selected protocol and trial information. Furthermore, the user can add semantic filters when searching for complete information to reduce the search scope or focus on certain areas of clinical trials. The data flow of the interface is illustrated in Fig 3.

The backend of the interface was developed using Node.js [52], and the visualization of the interface was manipulated by d3.js [53]. We developed custom functions on d3.js to present each element title and protocol count for the user’s selection. To implement semantic filtering of the user’s free-text input, a backend engine was connected to the representational state transfer (REST) NER API. This provided NER of processed entities and types, allowing relevant entities to be searched for in the database. We designed the system architecture to combine the interface application, APIs, and database for stable operation in a cloud-computing environment (S1 Fig).

## Results

### Database

We collected 184,634 clinical trial protocols and the frame structures of 13,210 clinical trial protocols from which we extracted 5,765,054 phenotypes, 1,151,053 chemical compounds, and 222,966 gene features for semantic filtering (Table 4). Furthermore, we designed a continuous process of data update so that the protocol methods could evolve naturally, thus enhancing the quality of the database (S2 Fig). In conclusion, we developed a database system that efficiently retrieves information about existing clinical trial protocols for use in designing new clinical trial protocols.

**Table 4:** Table schema for clinical trial protocol.

Entity	Rows (Unique)
Clinical Research Protocol	184,634 (184,634)
Frame Structures	13,210 (13,210)
Phenotype	5,765,054 (18,438)
Chemical Compounds	1,151,053 (12,792)
Genes	222,966 (4,705)

### Application

**Fig 4:**
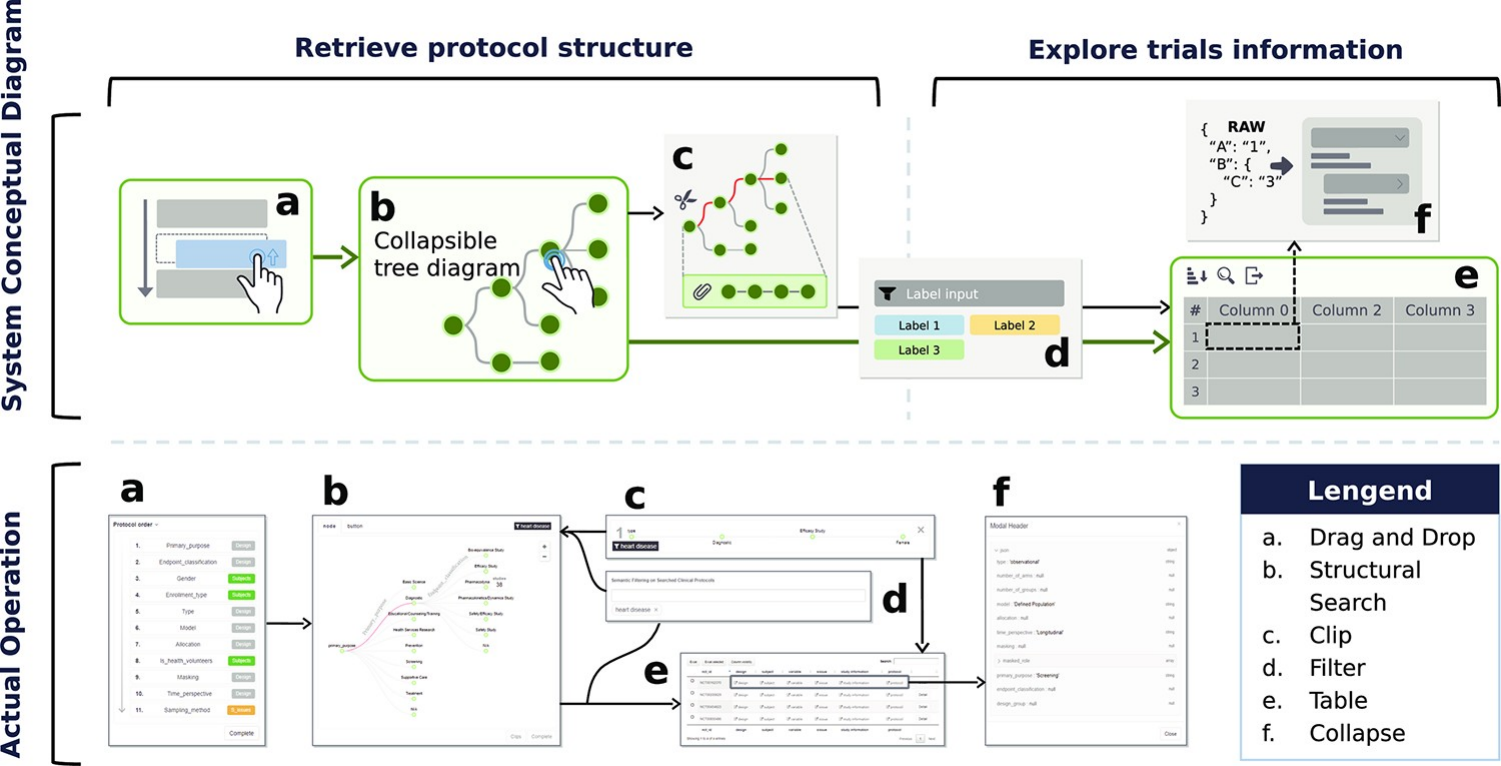
System overview.

The web application provides a service for retrieving protocol structures and inquiring about protocol information. The user traverses four stages when using this service: (1) setting the order of the protocols; (2) designing the protocol structure by selecting the elements that correspond to each sequence; (3) setting various functions required to search for the desired protocol information; and (4) providing the desired protocol information to explore the contents in detail. We developed the necessary interfaces to perform all of these processes (Fig 4).

Before retrieving the protocol structure, the user needs to define the protocol sequence. This process uses a drag-and-drop interface to sort the list into one box and set the order; it allows users to work more intuitively [54]. After determining the protocol sequence, the vector-based collapsible and zoomable tree diagram visualization interface, which is an uncomplicated tool, is used for navigating the protocol structures with the selected elements [55, 56]. The loaded protocol data are assembled into a hierarchical data structure. This visualization is rendered as a relation tree with a parent/child structure. The user clicks on the edge of the tree to add the next-step protocol. Conversely, to remove protocol edges from the current stage, the user must click on the parent edge of the previous step. The entire data structure is synchronized and updated every time the process occurs [57].

After the protocol structure has been retrieved, the user obtains protocol information based on the selected protocol structure. We developed a function called Clip to back up the protocol design. This allows the user to reuse previously selected protocol structures and receive corresponding study information. In addition, a protocol-information-filter function allows the user to retrieve the study information. The user searches for a disease and generates a label that contains the disease code. The protocol information is then filtered according to the set label. The resulting data are rendered as a table, which can be sorted with respect to the column entities to focus on and export specific data. When exploring detailed protocol information, our system transforms the data into a collapsible interface instead of providing raw text.

Although protocol design concepts have evolved globally, the development of tools to design clinical trial protocols is trivial [58–60]. Our aim was to simplify clinical trial retrieval and the design stage by developing a dedicated interface. We expect this to be the starting point for the creation, sharing, and development of more clinical trial protocols.

## Validation

### Technical validation

The goal of CLIPS was to provide a database system for information retrieval method that can search complex clinical trial protocols. To achieve this, we developed a search tool that can build and utilize a database suitable for protocol structures. Furthermore, we created semantic features in CLIPS using text-mining methods. As a result, it was possible to perform accurate searches using the protocol’s contextual meaning. To evaluate the performance of CLIPS, we attempted to verify whether the semantic filters perform better than a keyword search.

For technically validating the CLIPS’ semantic filter we used the relational information between clinical trial protocols and corresponding disease conditions as collected from clinicaltrials.gov [18]. As this disease condition assignment is manually curated by experts and does not originate from the protocol itself, it can be used as a gold standard to evaluate the semantic filters of CLIPS.

The gold standard set of disease conditions and corresponding trial protocols was obtained by crawling the topic page of clinicaltrials.gov [18]. Among 25 conditional categories provided by clinicaltrials.gov, the “Cancers and Other Neoplasms” condition category was selected as the first set because it covers 44.74% of the total protocol set. Consequently, the corresponding trial protocols and corresponding disease conditions were identified. For instance, in our gold standard set, 353 distinct protocols were associated with the disease condition “Abdominal neoplasm.” As a result, a set of 82,584 distinct protocols corresponding to 520 disease conditions was compiled (as of July 12, 2017) and used as a gold standard set for technical validation. In addition, we expanded the gold standard set to the rest of the 24 categories for sufficient validation. The expanded set has 289,956 distinct protocols corresponding to 6,172 disease conditions that were compiled (as of June 21, 2020). However, the collected set showed a 2.4% protocol loss from clinicaltrials.gov. This was occasioned by server overload during data crawling, and we omitted the loss.

The semantic search performance of CLIPS was validated using the following procedure. In CLIPS, search keywords that contained the disease condition’s nomenclature were supplied as input queries to the system. The semantic entities were translated from the search keyword through the text-mining-based models described in the previous section. The results were obtained by conducting a search using the translated semantic entities from the CLIPS database. Exact matching with the AACT database was used as a baseline. CLIPS and AACT databases were configured on a single local server.

We used a condition name (e.g., Adrenocortical Carcinoma) as a search keyword to retrieve the condition field of the source database and semantic entities (e.g., C0206686) from CLIPS. We then validated our retrievals by comparing the number of retrieved identifiers (NCTID) of protocols (S2 File) [61] and calculated the precision, recall, and F1-score of the results. Precision refers to how accurately the model is categorized and is calculated as by the ratio of properly categorized data to the total data (1). Recall is the number of positively classified data divided by the original number of positive data (2). The F1-Score is a harmonic mean that considers the complementary characteristics of precision and recall (3); it is commonly used to compare the performance of different information retrieval systems [62]. True positive + false positive is the count of all NCTIDs retrieved from each database. True positive is the count of the retrieved NCTIDs of each database intersected with the gold standard. True positive+ false negative is the total number of NCTIDs in the gold standard corresponding to each input condition name.

$$precision=\frac{true\;positive}{true\;positive+false\;positive}$$ (1)

$$recall=\frac{true\;positive}{true\;positive+false\;negative}$$ (2)

$$F1-Score=\frac{2*(recall*precision)}{\left(recall+precision\right)}$$ (3)

**Fig 5:**
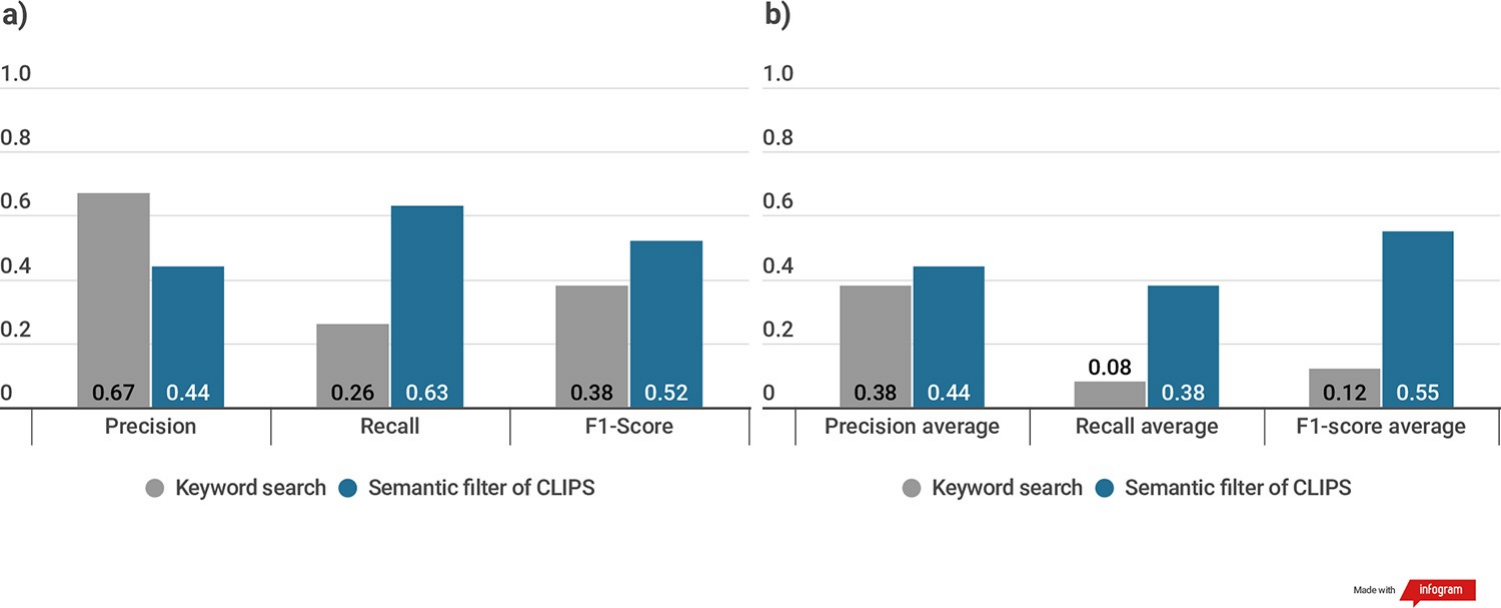
Evaluation results of keyword search and using semantic filter of CLIPS (a) Cancer and Other Neoplasms (b) The average values of the expanded 24 conditional categories.

The CLIPS’ F1-Score of CLIPS (0.515) was higher than that of the keyword search (0.38) (Fig 5A). The precision of CLIPS was 0.437, which was slightly lower than that of the keyword search (0.668), but it outperformed the keyword search by more than a factor of two in terms of recall (0.63 and 0.26, respectively). In the expanded conditional categories, the F1-Score of CLIPS (0.55) was higher than that of the keyword search (0.12) (Fig 5B). The precision of CLIPS was 0.44, which was slightly higher than that of the keyword search (0.39), but it also outperformed the keyword search by more than a factor of two in terms of recall (0.38 and 0.08, respectively). As higher recall values are a positive factor in clinical trial design, CLIPS can retrieve more protocols that provide more suitable references for protocol design.

**Fig 6:**
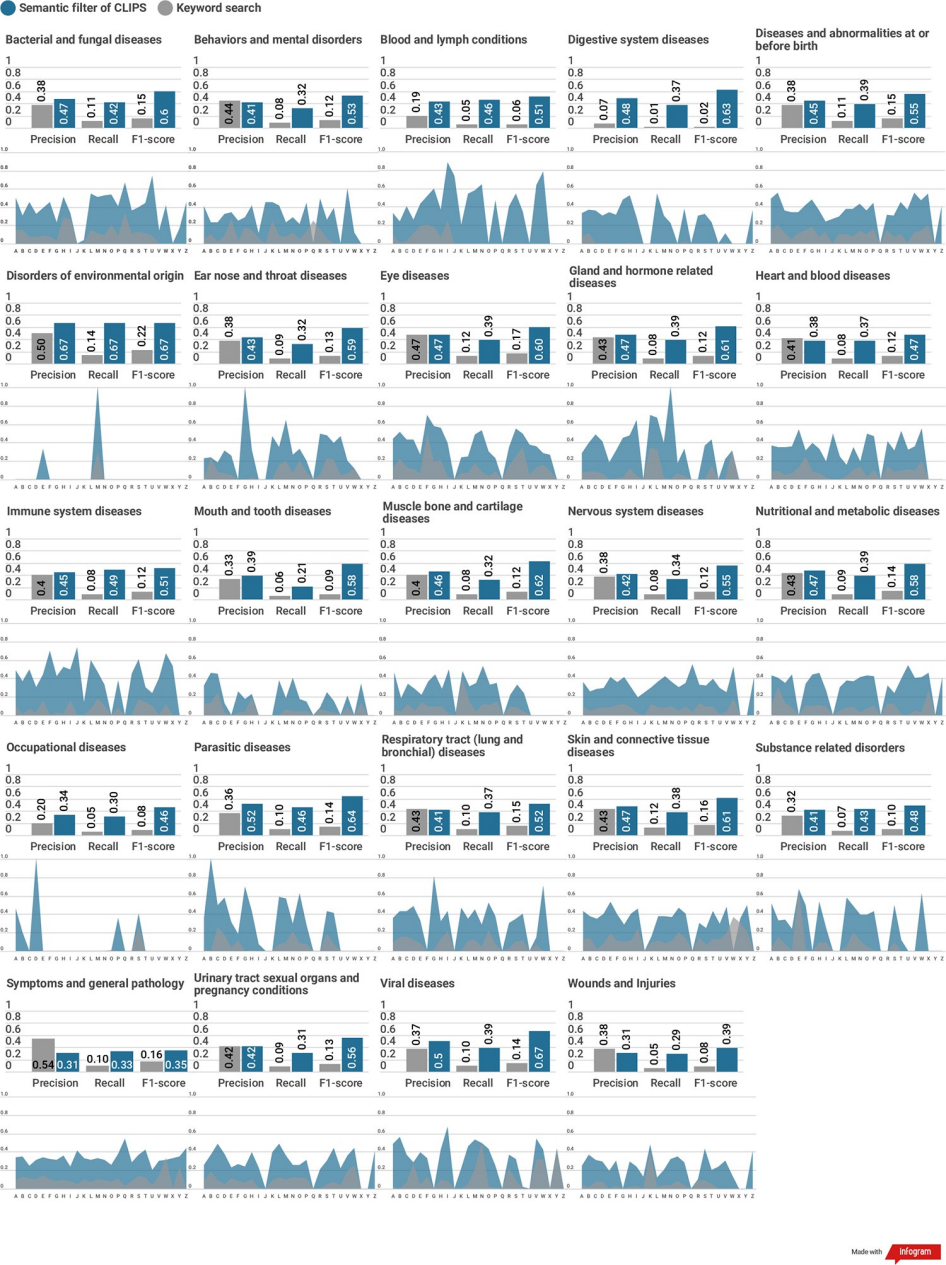
The detailed results of the keyword search and using the semantic filter of CLIPS on the expanded conditional categories.

Fig 6. shows the detailed scores of each expanded conditional category. It also shows that recall scores of each disease condition belong to a conditional category depicted as an area graph. Labels of the x-axis on the area graph are the initial character of each disease condition.

### User experience

As described earlier, the CLIPS search system was developed for different purposes than those of the existing clinical trial search systems. CLIPS is intended to assist in the effective design of a specific trial protocol, whereas existing search systems are generally used to process a variety of information on clinical trials. Therefore, to evaluate the performance of CLIPS, an evaluation method that reflects this purpose should be constructed. As the ultimate purpose of a search system is to help users collect the information they require, user’s satisfaction is a significant if subjective, measure of performance. Thus, the evaluation of a search system should be able to quantify the subjective impressions of users as well as objective indicators. By considering these factors, we conducted an evaluation trial that compared CLIPS with the conventional search system provided by clinicaltrials.gov.

**Fig 7:**
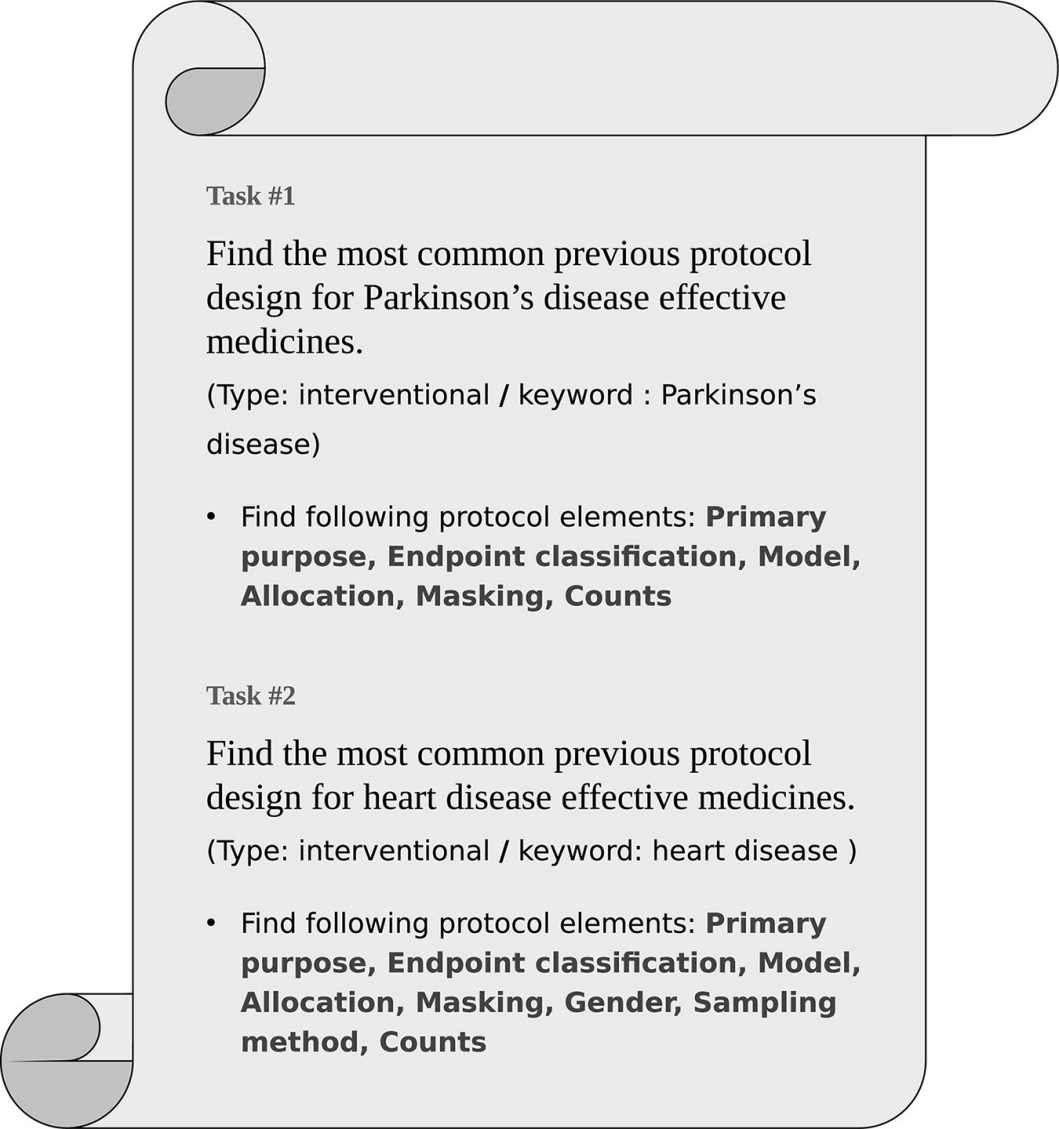
Tasks given to participants for evaluation.

Ten participants aged between 24 and 33 were recruited from a group of experts in the field of bioinformatics (Bio and Brain Engineering Department of Korea Advanced Institute of Science and Technology, Republic of Korea) They included two undergraduates, three master’s students, four Ph.D. candidates, and one postdoctoral researcher. All participants provided informed consent before conducting the evaluation trial. The participants were assigned the task of finding a suitable clinical trial set for a simulated problem. Two separate tasks were given to the participants, who were asked to construct the most common previous protocol design under the given trial conditions and research questions and perform each task using each of the two search systems within the time limit (5 min each). To construct the most common trial design, participants had to collect information about the various elements of the trial protocol (Fig 7). After the task, participants completed a questionnaire on their subjective satisfaction regarding the system. The questionnaire consisted of six questions that inquired about the participants’ satisfaction using a 7-point Likert scale [63].

Participants were observed to perform better when using CLIPS than when using the clinicaltrials.gov search system. By using CLIPS, participants obtained more answers within the time limit, and the average time required to perform the task was shorter than that with clinicaltrials.gov. The number of clinical trials retrieved from CLIPS was less than that from clinicaltrials.gov because it was possible to apply more detailed search filters to narrow the search scope. Participants were more satisfied with CLIPS than with the existing search systems, as evidenced by the average score of the questionnaire responses (Table 5). These results show that CLIPS can be effectively used to retrieve certain types of trial protocols.

**Table 5:** Evaluation results.

Measure (n = 10)	CLIPS		clinicaltrials.gov	
	Task1	Task2	Task1	Task2
Answer submitted, %	100.0	100.0	12.0	17.14
Elapsed time(minutes), mean(SD)	4.05 (0.86)	5.0 (0)	3.95 (0.89)	5.0 (0)
Count of retrieved trials by search, mean	137	179	1,591	8,484
How much do you think the retrieved result is suitable for the task? (range 1–7), mean (SD)	6.8 (0.18)		2.2(1.07)	
How much do you think you have had enough time to perform the task? (range 1–7), mean (SD)	6.6 (0.27)		1.2 (0.18)	
How difficult do you think it was to perform the task? (range 1–7), mean (SD)	1.6 (0.93)		6.2(1.07)	
How much do you trust the search result? (range 1–7), mean (SD)	6.2 (0.18)		3.6 (3.6)	
How satisfied are you with your answers based on your search results? (range 1–7), mean (SD)	6.4 (0.27)		1.8 (0.84)	
How satisfied are you with the search system? (range 1–7), mean (SD)	6.5 (0.5)		2.3 (0.68)	

## Discussion

Clinical trial protocols are the foundation for planning, approving, conducting, and reporting clinical trials [3]. They include general information, objectives, trial design, the selection and withdrawal of subjects, treatment, safety assessments, quality control procedures, and record-keeping processes [64]. This study aimed to develop an efficient system of providing the information necessary for clinical trial protocol development. In particular, we have made it possible to find previous protocols of the desired type using the structural features of the protocol composed of context-dependent protocol elements. Furthermore, semantic filtering was included to ensure the retrieval of relevant protocol context information.

CLIPS can search for protocols or specific disease names and structures. In addition, CLIPS can be used for a combination of structural searches, structural order searches, semantic searches, or searches including both structure and semantic context. For instance, our system can perform the following functions:

We suppose that a user has a plan to develop a clinical trial protocol about cardiovascular infections, and the user will perform an observational study and should decide the sampling method.

1. Input 'Cardiovascular infections' to the semantic filtering input box.
2. Order elements as follows: 1. Type, 2. Sampling method. The system orders the rest of the elements automatically.
3. Click 'complete' on the protocol order interface.
4. Select 'node' on the graph-based search interface.
5. Click 'type' and then wait until the next element result is provided.
6. Click 'observational', and then, the user can find 'Non-probability sample' is the sampling method to the specific disease.
7. Click 'Clips' below the graph-based search interface
8. Click clipped protocol structure, and then, the system provides detailed information of 6 searched protocols.
9. User can follow or download more detailed protocol information based on a detailed description of the searched protocols from the explanation interface such as model, enrollment type, time perspective, and outcome variables.

In accordance with the above results, we believe that many clinicians will be able to utilize our system to design more reliable clinical trial protocols. The developed semantic filter can be used to search for protocols and can be used for drug discovery using the retrieved protocols. CLIPS provides search results as a downloadable file containing the semantic filters as well as protocol structure information. This ensures wide protocol search coverage. For example, we used UMLS as a phenotype semantic filter. UMLS is an integrated terminology system that combines biomedical terminologies including SNOMED-CT, MeSH, and MedDRA [65]. Clinical terms in SNOMED CT have been integrated into the UMLS metathesaurus since 2003 [66]. For instance, coverage of the HPO term is higher than in SNOMED CT [67]. According to Bodenreider’s study, UMLS covered 54% of the HPO phenotype terms, whereas SNOMED CT covered only 30% [37]. Based on the semantic filter, researchers can screen chemical compounds of drug candidate substances, regardless of whether they are known to be effective, from the previously retrieved protocols [68]. Gene and phenotype can also be used for drug efficacy screening or efficacious drug combinations in massive biological networks using a similar approach [69–71]. Furthermore, users can download the entire database. The efficacy weight of edges can then be predicted, the predicted pathways validated in large-scale biological networks, and be utilized to find their maximum therapeutic benefits [72, 73].

The present study is limited in terms of user-experience validation. This is because clinicaltrials.gov and CLIPS have different objectives. Clinicaltrials.gov was developed to register clinical trials [18]. The registered information can be retrieved by clinical researchers, patients, and families of patients. This is a different objective from that of CLIPS, which is specially designed for the protocol search task. As the objective is different, the method is different. Therefore, it cannot be claimed that the clinicaltrials.gov offers worse performance than CLIPS, although the user-experience validation experiment showed a low score for use of clinicaltrials.gov. The satisfaction score of CLIPS is high in terms of the objective of protocol searching. If clinicaltrials.gov were to include the search function of CLIPS, it would offer clinical researchers the ability to search comprehensively for specific information.

Moreover, this study is the baseline step of a computerized clinical protocol development system. In future work, it would be promising to define the comprehensive similarity among the protocols and rank them based on semantic vector representations of each data feature of the protocols [74]. To validate the result, ranking evaluation models on information retrieval, such as MRR (mean reciprocal rank), nDCG (normalizing discounted cumulative gain), MAP (mean average precision) or Top-Kr recall methods should be used based on a consensus on the rank criteria [75].

## Conclusion

Clinical trial protocols are crucial for clinical trials to achieve their primary purposes. However, clinical researchers tend to design clinical trials according to their expertise. This type of bias or ambiguity may lead to inconsistencies and objectivity problems when clinical trial protocols are designed. To solve these problems, an information retrieval system for clinical trial protocols is needed. In this study, we developed a clinical trial protocol database system and a web application. The database contains design, subject, variable, statistical issue, description, and structure of clinical trial protocols. The web-based system provides a graph-based search interface based on the structure; users can then find relevant information on a protocol from the database. Furthermore, the database also includes semantic features to assist the context-specific protocol search. Unlike the previous clinical trial database system, our system has the following two main strengths: (i) it provides structural information to present simplified element-wise selection and (ii) extends the search field based on filterable semantic features to do a context-specific search for clinical trial protocols. We believe that CLIPS will be a major resource for clinical trials and will be of interest to clinicians and pharmaceutical companies or even regulatory agencies by providing information about clinical trial protocols, conveniently. This study has described the formulation of the CLIPS database system and explained its implementation and advantages over existing keyword-based search systems.

The whole database is available for download (http://corus.kaist.edu/clips).

## Supporting information

S1 File Supplementary Data 1. (XLSX)

S2 File Supplementary Data 2. (XLSX)

S3 File Supplementary Data 3. (CSV)

S4 File Supplementary File. (DOCX)

S1 Fig Example of factor, element, and value in the definition section. The “design” factor of a protocol includes elements, and among the elements, the “model” contains values. *E*_*n*_ is the number of elements in a factor; *V*_*n*_ is the number of values in an element. For example, The values consist of “Crossover Assignment” to “Case-only”, and the “model” element of the “design” factor has one of the values.
(PDF)

S2 Fig CLIPS service architecture on Amazon web service. Domain name service (DNS) uses the KAIST domain server to use kaist.edu. A user accesses the CLIPS service through the DNS. When accessing CLIPS through the DNS, the interface elastic compute cloud (EC2, https://aws.amazon.com/ec2/) is called, and it displays a screen to the user. Interface EC2 connects to API Engine EC2 to process the data requested by the user. If the user uses a semantic filter, API engine EC2 transfers the input value of the user to text mining EC2, and then, it receives the result. Particularly, text mining EC2 is composed of Metamap^1^, Moara^2^, and Chemspot^3^, Dockers^4^, which we customize for our service in the elastic container service (ECS, https://aws.amazon.com/ecs/) group. To provide the data requested by the user, API engine EC2 receives the searched result from the CLIPS relational database service (RDS, https://aws.amazon.com/rds/) in which the clinical trial protocol data are stored, and it transfers the result to interface EC2. Furthermore, we use elastic load balancing (ELB, https://aws.amazon.com/elasticloadbalancing/) for stable service traffic control, and ELB is required to make requests for the EC2 groups that are grouped into the auto scaling group (https://aws.amazon.com/ec2/autoscaling/).
(PDF)

S3 Fig Continuous data update flowchart of CLIPS. The DB Checker, a database change detection function based on the Quartz job scheduler (http://www.quartz-scheduler.org) built in the CLIPS API engine, operates as follows: (1) DB checker detects whether the source database is changed or not. (2) DB Checker detects whether schema of the database is changed or not. (3) Schema change (3–1). In case of schema change, the program cannot process it automatically. Therefore, it needs to analyze the data manually. The DB checker sends an update notification email to the CLIPS developers using the AWS simple notification service (https://aws.amazon.com/sns/). (4) Schema not changed. Only new data are added. (4–1) The modified dataset is stored in the CLIPS temporary data storage table. (4–2) Protocol structure information is extracted from a temporary data table. (4–3) Semantic features are extracted from the texts of the data using the CLIPS text mining engine. (4–4) The result obtained in the previous step is stored in the CLIPS database, and the update is completed.
(EPS)

S4 Fig CLIPS example of protocol structure retrieval by the selection of a categorical type element. A user selects the type as the first element in the categorical type element and then chooses the intervention category. The second categorical type element is of primary purpose, and the diagnostic is decided among the nine categories included in the element.
(PNG)
