# Supplementary material for: An interactive retrieval system for clinical trial studies with context-dependent protocol elements
Source: PLoS One. 2020 Sep 18;15(9):e0238290. doi: 10.1371/journal.pone.0238290 (PMC7500653; doi:10.1371/journal.pone.0238290)
Supplement: S4 File — (DOCX) [file pone.0238290.s004.docx]

**Supplementary File**

-----------------------------------------------------------------------------------------------------------------------------------------------

**An Interactive Retrieval System for Clinical Trial Studies with Context-Dependent Protocol Elements**

**Authors**

Junseok Park^1,2^, Seongkuk Park^3^, Kwangmin Kim^1,2^, Woochang Hwang^4^, Sunyong Yoo^5^, Gwansu Yi^1^ and Doheon Lee^1,2*^

^1^Department of Bio and Brain Engineering, Korea Advanced Institute of Science and Technology (KAIST), Daejeon 34141 Republic of Korea

^2^Bio-Synergy Research Center, KAIST, Daejeon 34141, Republic of Korea

^3^Information & Electronics Research Institute, Daejeon, 34141 Republic of Korea

^4^The Milner Therapeutics Institute, University of Cambridge, CB2 1QN United of Kingdom

^5^School of Electronics and Computer Engineering, Chonnam National University, Gwangju, 61186 Republic of Korea

***** Corresponding author

**Additional explanation**

**
Supplementary Figure 1. Example of factor, element, and value in the definition section.** The “design” factor of a protocol includes elements, and among the elements, the “model” contains values. *E_n_* is the number of elements in a factor; *V_n_* is the number of values in an element. For example, The values consist of “Crossover Assignment” to “Case-only”, and the “model” element of the “design” factor has one of the values.

**CLIPS system architecture**

**
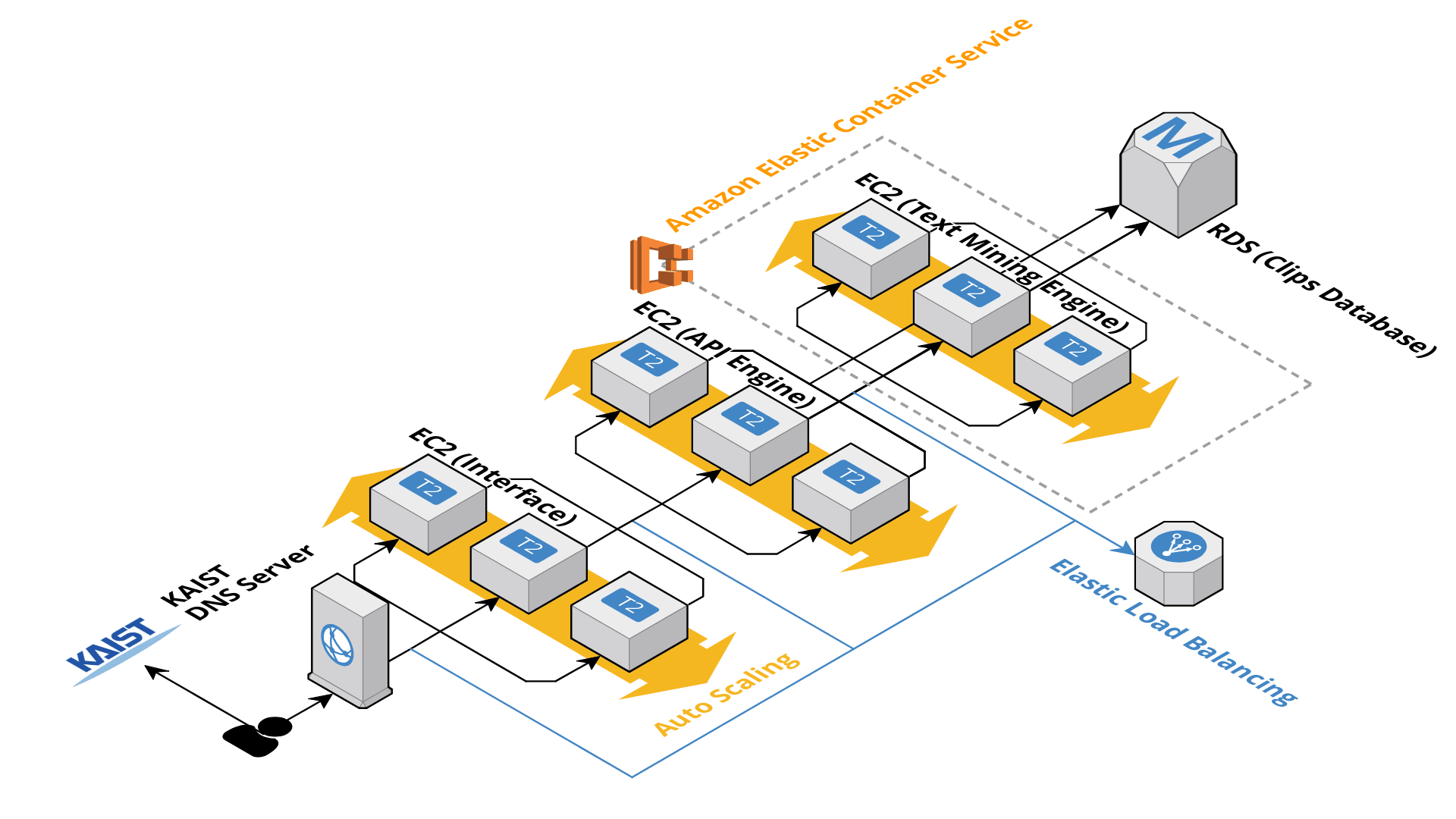

Supplementary Figure 2. CLIPS service architecture on Amazon web service.** Domain name service (DNS) uses the KAIST domain server to use kaist.edu. A user accesses the CLIPS service through the DNS. When accessing CLIPS through the DNS, the interface elastic compute cloud (EC2, https://aws.amazon.com/ec2/) is called, and it displays a screen to the user. Interface EC2 connects to API Engine EC2 to process the data requested by the user. If the user uses a semantic filter, API engine EC2 transfers the input value of the user to text mining EC2, and then it receives the result. Particularly, text mining EC2 is composed of Metamap^1^, Moara^2^, and Chemspot^3^Dockers^4^, which we customize for our service in the elastic container service (ECS, https://aws.amazon.com/ecs/) group. To provide the data requested by the user, API engine EC2 receives the searched result from the CLIPS relational database service (RDS, https://aws.amazon.com/rds/) in which the clinical trial protocol data are stored, and it transfers the result to interface EC2. Furthermore, we use elastic load balancing (ELB, https://aws.amazon.com/elasticloadbalancing/) for stable service traffic control, and ELB is required to make requests for the EC2 groups that are grouped into the auto scaling group (https://aws.amazon.com/ec2/autoscaling/).

**Plan to ensure that the data in the database are current**

Continuous database updates have been considered since CLIPS was developed. To do this, we developed an API engine to import and update the data from the source databases^5,6^. The first step was to import the oracle dump file provided by the AACT^5^ database (https://www.ctti-clinicaltrials.org/aact-database) into the Docker^4^ container and then extract the necessary data into the CLIPS database. The API engine of CLIPS used jsoup (https://jsoup.org/) to notify us by e-mail when the web page providing the data was updated. We planned to update it manually. However, while we were developing CLIPS, a major change (https://www.ctti-clinicaltrials.org/aact-database) occurred in the source database, and the original development version had to be updated. Therefore, our future plan is as follows: We will update the existing functionality. The function will monitor the changes directly by connecting to the source database platform (https://aact.ctti-clinicaltrials.org/psql). If changes are made, we will update the CLIPS database by extracting the new clinical trial protocol information according to the type of change. Details of the plan are described in Supplementary Figure 2.

**
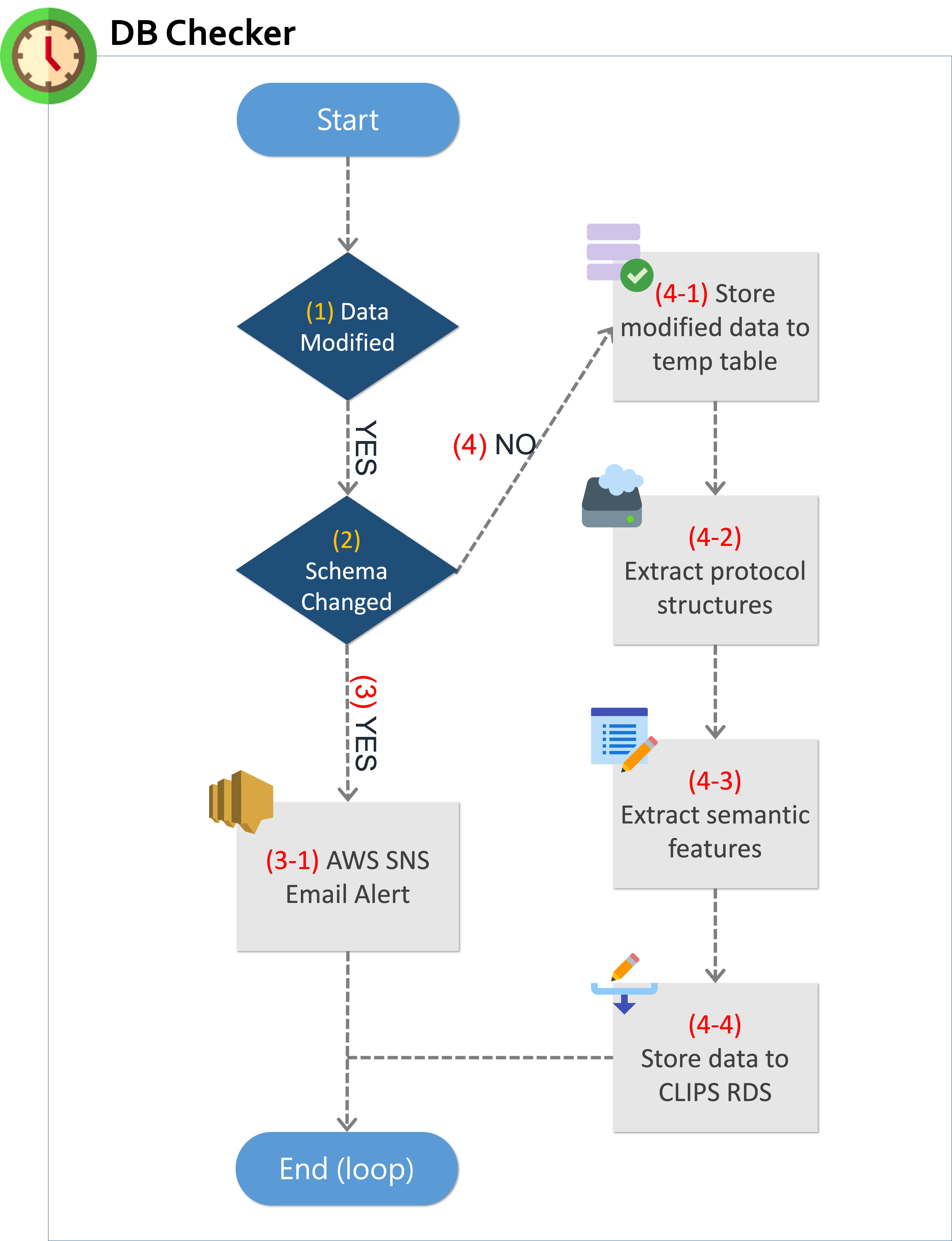

Supplementary Figure 3. Continuous data update flowchart of CLIPS. The** DB Checker, a database change detection function based on the Quartz job scheduler (http://www.quartz-scheduler.org) built in the CLIPS API engine, operates as follows: (1) DB checker detects whether the source database is changed or not. (2) DB Checker detects whether schema of the database is changed or not (3) Schema change (3-1) In case of schema change, the program cannot process it automatically. Therefore, it needs to analyze manually. The DB checker sends an update notification email to the CLIPS developers using the AWS simple notification service (https://aws.amazon.com/sns/). (4) Schema not changed. Only new data are added. (4-1) The modified dataset is stored in the CLIPS temporary data storage table. (4-2) Protocol structure information is extracted from a temporary data table. (4-3) Semantic features are extracted from the texts of the data using the CLIPS text mining engine. (4-4) The result obtained in the previous step is stored in the CLIPS database, and the update is completed.

**Additional commentary of Categorical variable**

Categorical variables can be called nominal variables. Categorical variables can be categorized into more than one category and have no intrinsic ordering. We have classified the elements of the protocols as categorical type elements if they satisfy the definition of a categorical variable. The categorical type elements can be further classified according to the key factors to which they originally belong: design, subject, variable, and statistical issue (Supplementary Data 2). For example, a type belonging to the design factor is a categorical type element, which has two categories (interventional and observational). The structure of the protocol is determined by the sequential decision of the categorical type elements belonging to the key factors (Supplementary Figure 3).


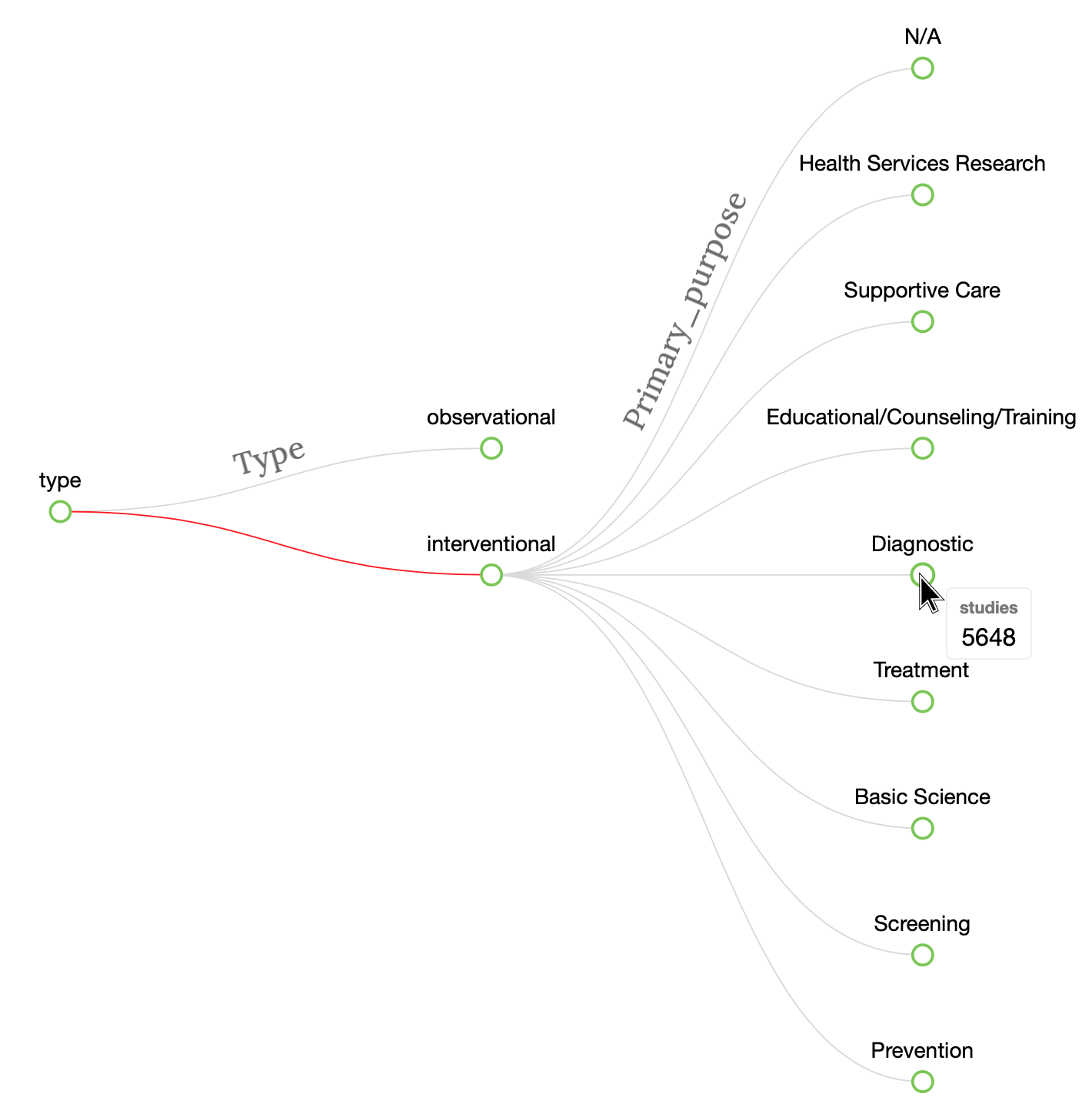

**Supplementary Figure 4. CLIPS example of protocol structure retrieval by the selection of a categorical type element.** A user selects the type as the first element in the categorical type element and then chooses the intervention category. The second categorical type element is of primary purpose, and the diagnostic is decided among the nine categories included in the element.

**References**

1 Aronson, A. R. J. B., MD: NLM, NIH, DHHS. Metamap: Mapping text to the umls metathesaurus. 1-26 (2006).

2 Neves, M. L., Carazo, J.-M. & Pascual-Montano, A. Moara: a Java library for extracting and normalizing gene and protein mentions. *BMC Bioinformatics***11**, 157, doi:10.1186/1471-2105-11-157 (2010).

3 Rocktäschel, T., Weidlich, M. & Leser, U. ChemSpot: a hybrid system for chemical named entity recognition. *Bioinformatics***28**, 1633-1640, doi:10.1093/bioinformatics/bts183 (2012).

4 Merkel, D. Docker: lightweight linux containers for consistent development and deployment. *Linux Journal***2014**, 2 (2014).

5 Tasneem, A. *et al.* The database for aggregate analysis of ClinicalTrials. gov (AACT) and subsequent regrouping by clinical specialty. *PloS one***7**, e33677 (2012).

6 Zarin, D. A. & Keselman, A. Registering a clinical trial in ClinicalTrials. gov. *CHEST Journal***131**, 909-912 (2007).
