# Supplementary figures and images for: An interactive retrieval system for clinical trial studies with context-dependent protocol elements
Source: PLoS One. 2020 Sep 18;15(9):e0238290. doi: 10.1371/journal.pone.0238290 (PMC7500653; doi:10.1371/journal.pone.0238290)

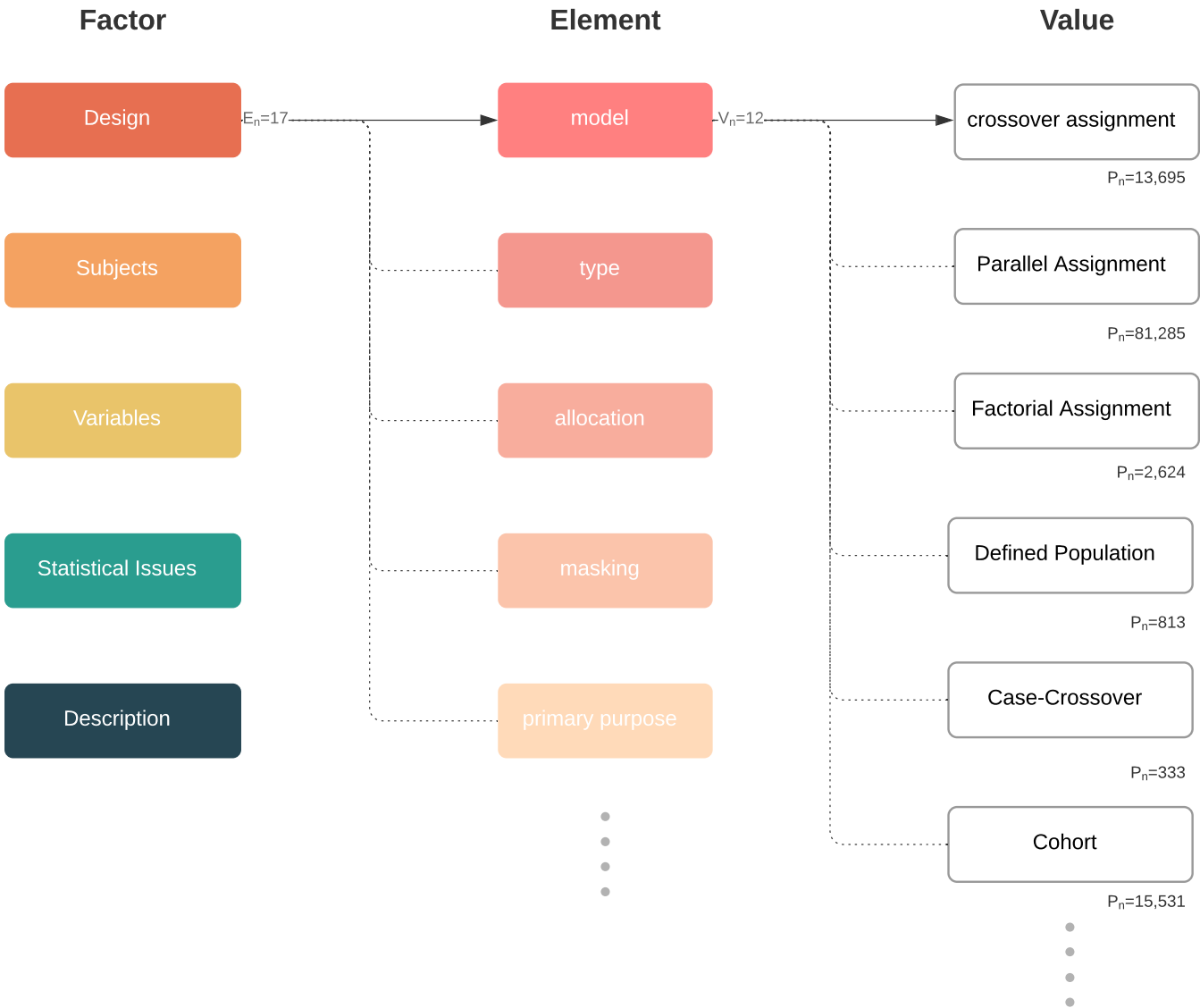

Supplement: S1 Fig — The “design” factor of a protocol includes elements, and among the elements, the “model” contains values. En is the number of elements in a factor; Vn is the number of values in an element. For example, The values consist of “Crossover Assignment” to “Case-only”, and the “model” element of the “design” factor has one of the values. (PDF) [file pone.0238290.s005.pdf]

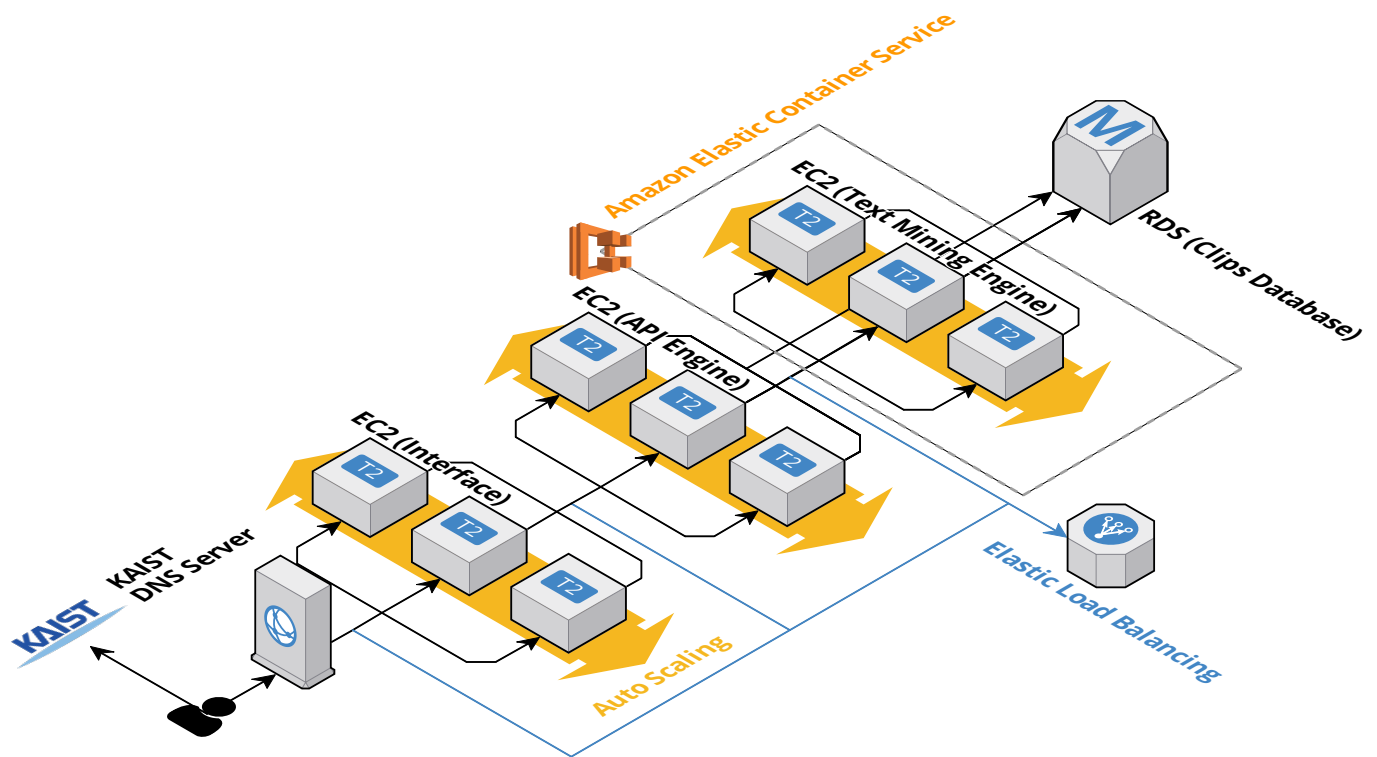

Supplement: S2 Fig — Domain name service (DNS) uses the KAIST domain server to use kaist.edu. A user accesses the CLIPS service through the DNS. When accessing CLIPS through the DNS, the interface elastic compute cloud (EC2, https://aws.amazon.com/ec2/) is called, and it displays a screen to the user. Interface EC2 connects to API Engine EC2 to process the data requested by the user. If the user uses a semantic filter, API engine EC2 transfers the input value of the user to text mining EC2, and then, it receives the result. Particularly, text mining EC2 is composed of Metamap1, Moara2, and Chemspot3, Dockers4, which we customize for our service in the elastic container service (ECS, https://aws.amazon.com/ecs/) group. To provide the data requested by the user, API engine EC2 receives the searched result from the CLIPS relational database service (RDS, https://aws.amazon.com/rds/) in which the clinical trial protocol data are stored, and it transfers the result to interface EC2. Furthermore, we use elastic load balancing (ELB, https://aws.amazon.com/elasticloadbalancing/) for stable service traffic control, and ELB is required to make requests for the EC2 groups that are grouped into the auto scaling group (https://aws.amazon.com/ec2/autoscaling/). (PDF) [file pone.0238290.s006.pdf]

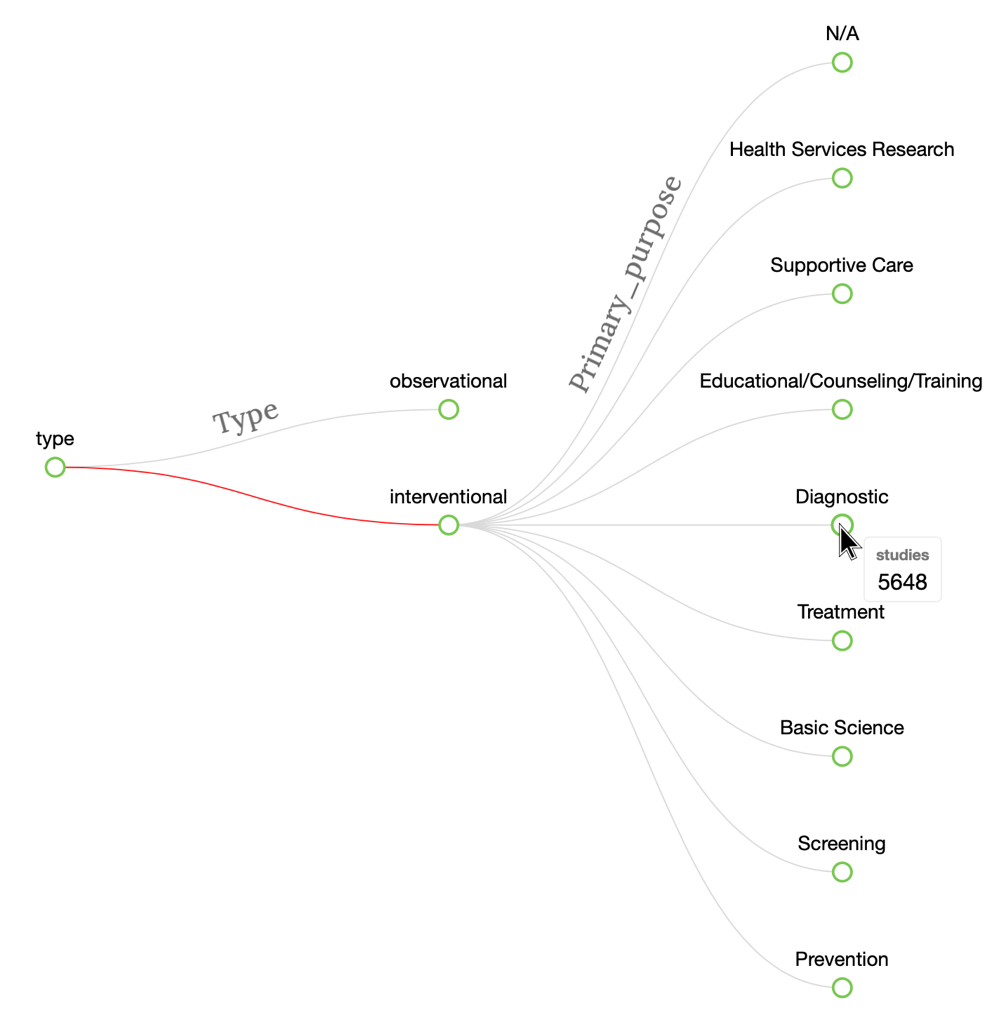

Supplement: S4 Fig — A user selects the type as the first element in the categorical type element and then chooses the intervention category. The second categorical type element is of primary purpose, and the diagnostic is decided among the nine categories included in the element. (PNG) [file pone.0238290.s008.png]
